# Evolution in a Changing Environment

**DOI:** 10.1371/journal.pone.0052742

**Published:** 2013-01-10

**Authors:** Andrea Baronchelli, Nick Chater, Morten H. Christiansen, Romualdo Pastor-Satorras

**Affiliations:** 1 Laboratory for the Modeling of Biological and Socio-technical Systems, Northeastern University, Boston, Massachusetts, United States of America; 2 Behavioural Science Group, Warwick Business School, University of Warwick, Coventry, United Kingdom; 3 Department of Psychology, Cornell University, Ithaca, New York, United States of America; 4 Santa Fe Institute, Santa Fe, New Mexico, United States of America; 5 Departament de Fisica i Enginyeria Nuclear, Universitat Politecnica de Catalunya, Barcelona, Spain; University of Zaragoza, Spain

## Abstract

We propose a simple model for genetic adaptation to a changing environment, describing a fitness landscape characterized by two maxima. One is associated with “specialist” individuals that are adapted to the environment; this maximum moves over time as the environment changes. The other maximum is static, and represents “generalist” individuals not affected by environmental changes. The rest of the landscape is occupied by “maladapted” individuals. Our analysis considers the evolution of these three subpopulations. Our main result is that, in presence of a sufficiently stable environmental feature, as in the case of an unchanging aspect of a physical habitat, specialists can dominate the population. By contrast, rapidly changing environmental features, such as language or cultural habits, are a moving target for the genes; here, generalists dominate, because the best evolutionary strategy is to adopt neutral alleles not specialized for any specific environment. The model we propose is based on simple assumptions about evolutionary dynamics and describes all possible scenarios in a non-trivial phase diagram. The approach provides a general framework to address such fundamental issues as the Baldwin effect, the biological basis for language, or the ecological consequences of a rapid climate change.

## Introduction

The issue of the evolution and adaptation in a changing environment has recently become hotly debated in the context of climate change and its effects on the extinction rates and the alteration of the distribution of species [1]–[4], but it is crucial in many domains. A classical example is given by lactose tolerance, where the advent of dairying created an environmental pressure in favor of this genetic trait, that in turn further increased the benefit of dairying, in a positive feedback loop [5]. Furthermore, it has been argued that this so-called Baldwin effect [6] may apply to many other aspects of the human evolution, such as the evolution of a language faculty. Here, an established linguistic convention would create a selective pressure, enhancing the reproductive fitness of those individuals that happen by chance to learn it faster or better. Over time, less environmental exposure would therefore be needed and what was originally a linguistic convention would eventually become encoded in the genes of the whole population [7]. On the other hand, it has been argued that language is a moving target which changes too rapidly for genes to follow [8]. This paper provides an analytical framework for addressing these issues.

The study of evolution and adaptation in a fluctuating environment from the perspective of population genetics has been hampered by the mathematical difficulty of the problem [9]. Various modeling attempts have focused on the conditions leading to adaptation, or lack thereof, from the perspective of the Baldwin effect (e.g. [10], [11]), or on species distributions in the presence of environmental stresses (e.g. [12]–[14]), but a general picture is still lacking. These models, in fact, while useful and interesting, are usually defined in terms of a large number of parameters, which compromise the possibility of studying them thoroughly from an analytical point of view, or of achieving fundamental insights into the problem at hand. Other work has instead followed the quasi-species approach [15]–[18], but also these models are in general characterized by considerable mathematical complexity.

Here we propose a stochastic interacting particle model that captures the most basic features characterizing evolution in a dynamic environment. The model is simple and analytically tractable at a mean-field level, and provides a general view of the gene-environment dynamics. Our work follows the statistical physics approach to evolutionary dynamics, which has become increasingly influential [19]–[22], clarifying, for example, crucial issues such as the role of the topology defining the interaction patterns in an evolving population [23] or system-size effects [24].

The model describes a large population of 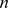 individuals and an external, environmental feature. Individuals are divided in three general types: “specialists” who are adapted to the environment, “generalists” whose fitness is independent of the environment, and “maladapted.” We assume that a complex network of genes codes for the ability to adapt to a specific feature of the environment. For example, we might focus on the linguistic environment, which has many specific forms corresponding to particular languages, such as English. A perfect tuning of this network would allow a “specialist” individual to learn very rapidly the specific language for which it is optimized, thus increasing her fitness (ability to survive and reproduce). Here we can say that the genes are aligned with a specific feature of the environment [11]. However, specialization comes with a cost, reducing the flexibility of the specialized genome [25]. Even a slight environmental change might cause problems to the offspring inheriting a genetic machinery evolved for the original, but now different, environment. The new individual would in fact be *mis*aligned (maladapted) and her fitness would be lowered. For this reason we include in the model also a third kind of genomes, namely the neutrals (or “generalists” [26]), for which the fitness of an individual is independent of the specific environment. Note that working with just one environmental feature corresponds to the assumption, standard when modeling complex biological phenomena [27], that different features of the environment have roughly independent impacts on fitness.

The dynamics of the model is defined in terms of evolutionary rules that depend on three basic parameters: The genetic mutation rate 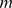, the rate of environmental change 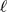, and a parameter 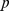, indicating the probability that an environmental change could lead to conditions favorable for previously maladapted individuals. As we will see, the probability 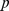 turns out to induce only small corrections in the biological limit 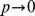. In terms of the remaining parameters 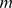 and 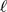, a non-trivial mean-field phase diagram can be drawn, exhibiting different phase transitions, akin to the so-called “error catastrophe” [28], as a function of 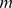 for small and large values of the rate of environmental change. This phase diagram describes the general conditions for microevolutionary adaptation in the presence of environmental stresses, and explains different empirical observations of adaptation in changing environments in a single framework.

## Results

### Model definition

The model is defined in terms of three different types, namely 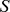, 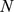, and 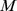, that represent Specialized, generalist/Neutral, and Maladapted individuals, respectively. Our aim is to describe a population in which the environment changes. Thus, thinking in terms of a theoretical fitness landscape [29], we assume that it exhibits a maximum whose position changes whenever there is an environmental variation, i.e., the maximum represents a *moving target*. The main simplification of our model, as opposed to standard quasi-species approximations [15], [18], consists in considering the class of specialists 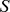 not as a *fixed* genome, but as the *set* of those genomes which are closer to the maximum of the fitness landscape, whatever the position of this maximum might be. In this theoretical fitness landscape we assume also the presence of a secondary, local, maximum, of lower height, representing the neutral genomes which are not affected by environmental changes. The position of this secondary maximum is considered static, since neutrals do not react to changes in the environment. From this perspective, our model borrows from quasi-species models in multiple-peaked landscapes [30], with the proviso that the absolute maximum moves in time, and we do not focus on fixed genomes, but on the set of those close to the maxima.

**Figure 1:**
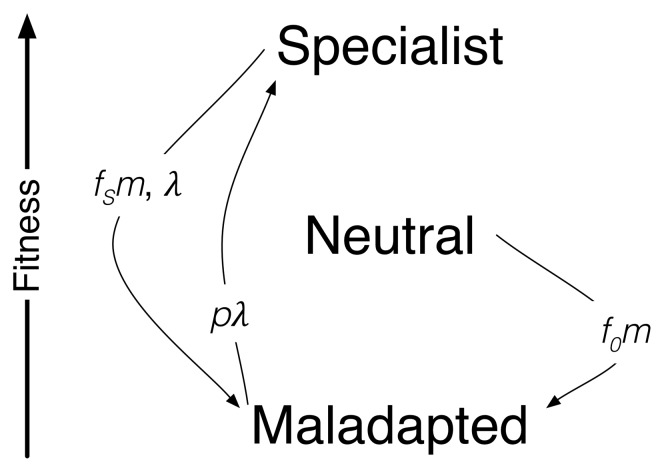
Schematic representation of the model definition. In reproduction, the number of offspring is, of course, proportional to the fitness of the parent genome. Genetic mutations happen with probability 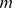, and are detrimental, and leading to offspring in the maladapted class. Environmental changes occur with probability 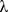, independently from the state of the population. Such changes typically damage specialists, but also favor previously maladapted individuals. The latter case is however less frequent, and it is therefore modulated by a further probability 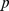.

In mathematical terms, the class 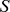 can thus be described as the set of genomes that are close to the principal maximum, by a distance 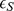. Analogously, species 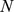 represents the set of genomes close to the perfectly neutral genome (the secondary fixed maximum), by a distance 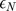. Finally, the set 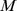 is composed of the remaining possible genomes. We assume a haploid reproduction system, with a fitness for each class 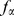, satisfying the restriction 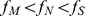. We consider these fitnesses as constant, independent of the environmental changes. At a mean-field level, assuming homogeneous mixing, the dynamics of the model is defined as follows (see Fig. 1): Reproduction is performed by selecting an individual with probability proportional to its fitness, as in standard haploid models (i.e., the Moran process [31]). The individual then produces an offspring which is equal to itself with probability 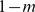, and that mutates to a different type with probability 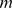. Conservation of individuals is achieved in reproduction by eliminating a randomly chosen individual. Crucially, all genetic mutations are assumed to be harmful, because the probability that they will lead to an increase of fitness is negligible [32]. Therefore, a genome of type 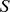 or 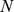, when mutating, reproduces into a type 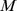, while 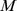 genomes always reproduce into 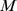 individuals. Environmental changes correspond to a shift of the position of the principal maximum of the fitness landscape. This shift is assumed to take place at each time step with a small probability 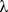, and produces different effects on the three species 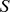, 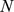 and 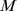. Specifically, a changing environmental does not, by assumption, affect the neutrals 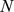. But the shift is mainly unfavorable to 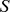 individuals, which were best adapted to the previous position of the maximum. This effect is implemented by selecting, with probability 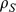, a specialized individual that will become maladapted, i.e., of class 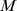. Finally, the shift could have a beneficial effect on other previously maladapted individuals, who were, in genomic space, far from the previous position of the principal maximum but are now close to its present one. This effect, which we assume to be rarer, is implemented by choosing with a small probability 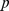 a maladapted individual (with probability 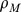), which will become specialized. Note that we neglect backward genetic mutations from 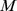 genomes to either 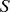 or 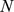 species. Thus, we are considering the most common scenarios in which beneficial mutations are much less frequent than harmful ones (see for example [33], [34]). Moreover, from the rules of the model, the population size 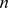 is constant. This restriction is not problematic for our purposes, since we are interested only in the ratios between the population densities of the different species. In what follows, however, we will consider the limit of an infinite population, 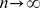, so that the presence of 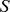 and 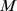 individuals can be assumed to be non-zero at the outset due to generic variability in the population, even thought they may be few in number. As we will see, the solution to our equations does not depend on the initial fractions of the different genomes.

### Mean-field rate equations

Let us define 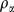 as the density of individuals in state 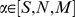, satisfying the normalization condition 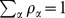. At the mean-field level, disregarding spatial fluctuations and stochastic fluctuations, and in the limit of 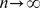, a mathematical description of our model can be readily obtained in terms of rate equations for the variation of the densities 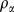. To construct those, we consider that a genome 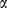 increases its number (*i*) when an individual 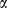 is chosen for reproduction and her offspring replaces an individual belonging to a genome 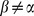, without any mutation, i.e., with probability 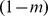, or (*ii*) when a mutation event leads an individual with genome 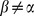 to reproduce into 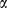. Conversely, a genome 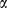 decreases when one individual belonging to it is randomly selected for replacement. Additionally, 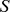 genomes may decrease their number due to a damaging change of the environment, while they can increase their number through a (rarer) beneficial environmental change. The corresponding rate equations take thus the form, writing explicitly all contributions to the change to each 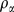,
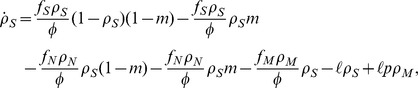


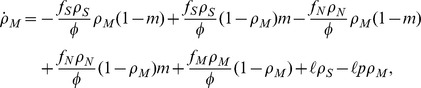


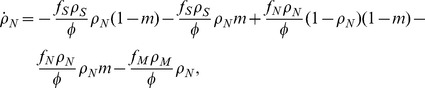
where we have defined *rate* of environmental change 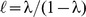, the average fitness of the population 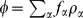, and we have performed an irrelevant rescaling of units of time. After some algebraic manipulations, the previous equations can be simplified to the form:
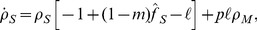
(1a)

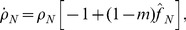
(1b)

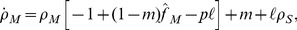
(1c)where we have defined 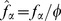.

[Disp-formula pone.0052742.e067] [Disp-formula pone.0052742.e068] Equations (1) completely define the dynamics of the model at the mean field level. In the following we will analyze their solution in different limits.

### Analytical solution

In the long term steady state, the solutions of this dynamical system are obtained by imposing the conditions 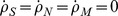 on [Disp-formula pone.0052742.e067] [Disp-formula pone.0052742.e068] Eqs. (1), solving the ensuing algebraic equations, and checking for the stability of the solutions, by looking at the eigenvalues of the Jacobian matrix, evaluated at the respective solution. The solutions obtained this way in the general case 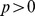 turn out to be quite complex, so in order to simplify the resulting expressions, we choose particular values of the fitnesses, namely 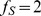, 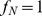 and 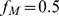, respecting their natural ordering. Solutions for other values can be obtained using the same steps.

#### Case 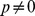


The algebraic equations ruling the steady state, obtained from [Disp-formula pone.0052742.e067] [Disp-formula pone.0052742.e068] Eqs. (1) by setting to zero the time derivatives, can be solved using a standard computational software package. This results in three sets of solutions, taking the form
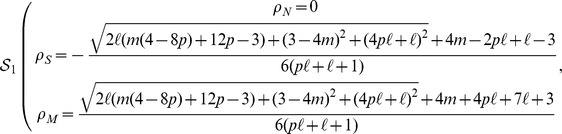


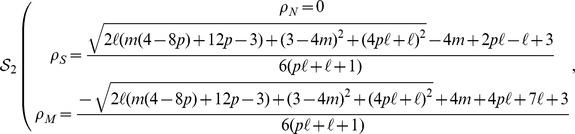


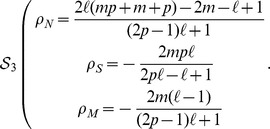



Solution 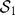 describes a 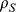 density that is negative in the parameter region 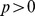, i.e., it is an “unphysical” solution that does not describe any realistic scenario. Solution 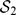 has nonzero densities in the whole parameter space, while solution 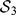 is physical only in the region
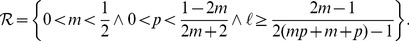
(2)In order to find the relative stability of the physical solutions 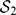 and 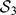, we consider the Jacobian matrix of the equation system [Disp-formula pone.0052742.e067] [Disp-formula pone.0052742.e068] Eqs. (1), taking the form
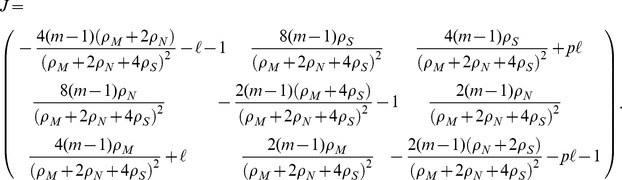
We then compute the eigenvalues of matrix 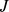, evaluated for the different solutions 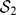 and 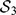. In any given region of parameter space, the stable solution (in the stationary limit) is the one possessing a negative largest eigenvalue. Examination of these eigenvalues, operation performed again with the help of standard computational software packages, leads to the solution:

- If 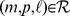:
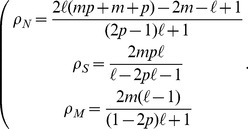
(3)
- Otherwise:
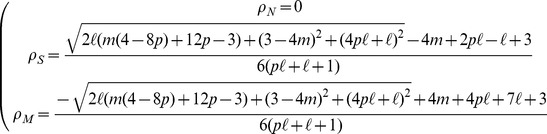
(4)where 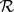 is the domain in the parameter space defined in Eq. (2).

**Figure 2:**
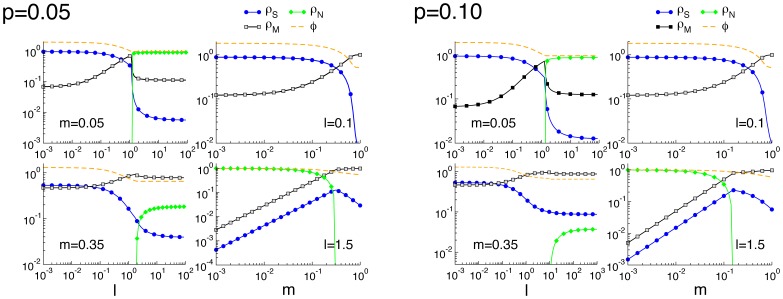
Species densities at the steady state for small 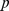
 **.** Densities of 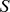 (blue), 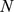 (green) and 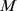 (gray) genomes as a function of the environmental mutation rate 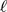 for fixed 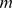, and as a function of 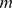 for fixed 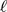. The left panel corresponds to 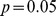, and the right panel to 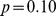. Dashed orange lines represent the average fitness of the population.

**Figure 3:**
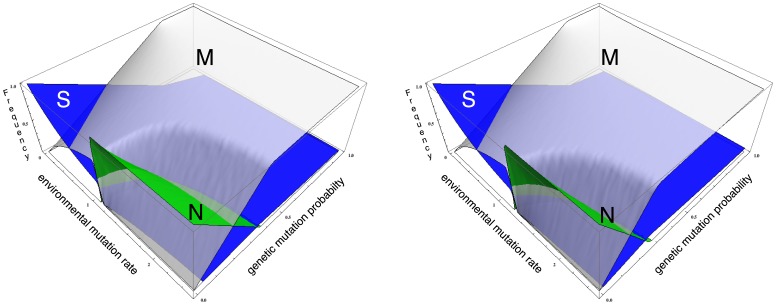
Species densities at the steady state for small 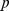
 **.** Densities of 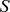 (blue), 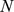 (green) and 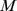 (gray) genomes as a function of the environmental mutation rate 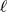 and the genetic mutation rate 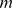, for fixed values 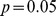 (left) and 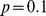 (right). Fitness values are 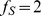, 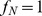 and 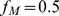.

The analytical solutions given by Eqs. (3) and (4) are quite complex, and it is difficult to extract direct interpretations from them. However, the behavior of the solutions can be understood in the biologically relevant region of small 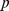. Fig. 2 shows the densities 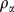 as a function of 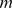 or 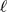, at fixed 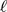 and 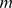 respectively, for two values of 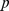, along with the value of the total fitness 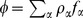. In the upper left corner plot for each value of 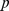, we consider the case 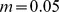, representative of the biologically relevant scenario of small mutation rate. When 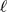 is very small, most of the population stays aligned with the environmental feature and 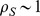. As 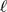 increases, maladapted genomes appear and eventually overcome the specialized genes. At a definite value of 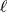, however, a discontinuity takes place and neutral individuals suddenly appear and become the majority of the population, while the density of both maladapted and specialists decreases. The decrease in specialists is larger for larger values of 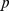. Thus, for sufficiently large 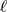 and small 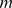, trying to catch up with the rapidly evolving environmental feature is not a viable strategy, since the risk of producing a maladapted offspring becomes destructive. Interestingly the strategy adopted by the majority of the individuals guarantees the maximum average fitness in any given region of the 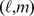 plane, for every value of 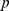. For large values of 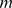 (lower left plots), the situation is qualitatively different. For small 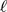, maladapted and neutral individuals are almost equally numerous. When 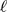 increases beyond a threshold, neutral individuals again appear suddenly, but they are unable to overcome maladapted genomes. Only for large values of 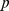 are neutrals capable to prevail over the specialists. The right plots for each value of 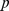 in Fig. 2 show the evolution of the species' densities as a function of 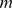 for fixed 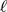. In this case, for small 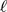 neutrals are absent from the system, and there is a simple competition between specialists and maladapted, the former being predominant for small genetic mutation rates, but going extinct for large 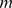. On the other hand, when the rate of environmental change is sufficiently large, we enter a new scenario in which neutrals are predominant for small 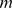. Beyond a mutation rate threshold, however, neutrals suddenly become extinct, and their population is replaced by maladapted genomes, while specialists decrease their density for large 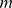. Interestingly, in this region of large 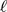, specialists can survive even for very large mutation rates, close to 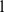, due to the effect of a nonzero 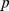 that prevents their complete elimination. Fig. 3 shows the complete picture of the relative species' abundances as as a function of 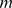 and 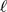 for the previously considered values of 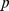.

#### Case 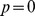


A more precise mathematical characterization of the phenomenology discussed above, and in particular of position of the transitions taking place for different values of 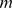 and 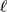, can be obtained in the particular case 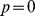. Here, qualitative arguments allow us to solve the model in a much simpler way, for general values of 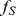, 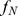 and 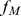. This analysis, moreover, reveals the role of 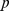 in the dynamics of our model.

The relevant equations in the 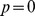 case read
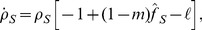
(5a)

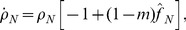
(5b)

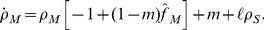
(5c)To find their solution, we argue as follows: If 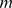 is very close to 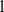, all terms in square brakets in [Disp-formula pone.0052742.e162] [Disp-formula pone.0052742.e163] Eqs. (5) will be negative. Therefore, the only stable solution will be 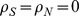, 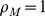, for any value of 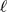. Under this conditions, the quantities within square brackets in Eqs. (5a) and (5b) take the form 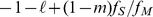 and 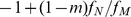, respectively. Decreasing the value of 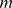, the first solution with 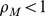 will take place for the first of these values that become zero. This occurs when 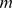 is smaller than either 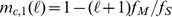 or 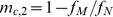, respectively. Since 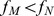, we have 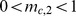, and this transition will always be physical. However, for 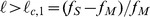, we have 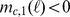 and it is not physical. In this case, when 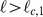, if 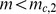, 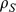 decays exponentially, and in the long time limit 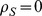; the existence of a non-zero 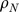 solution imposes 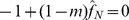, or 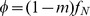, from where the restricted normalization condition 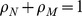 leads to the solution 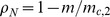 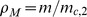, and 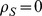. In the case 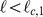, which density 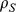 or 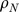 becomes first non-zero depends on which threshold, 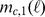 or 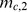 is larger. Thus, if 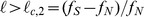, then 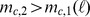. Therefore, when decreasing 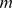, the first density to take a non-zero value is 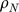. 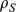 decays again exponentially, so the solution is the same as in the case 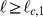. Finally, for 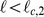, 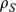 is the first density to become non-zero when 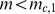. The steady state solution comes from imposing 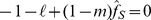, leading to 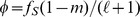. In this region, the factor in square brackets in Eq. (5b) becomes negative, indicating an exponential decay and a corresponding steady state value 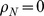. We are therefore led to the solution, using the normalization condition, 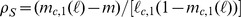, 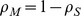.

The final solution in this case can thus be summarized as follows:

- For 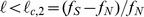

  If 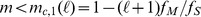


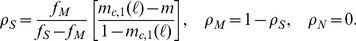
(6)
  If 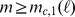


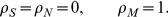
(7)
- For 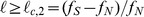:
  If 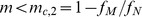


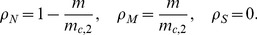
(8)
  If 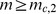


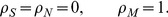
(9)

**Figure 4:**
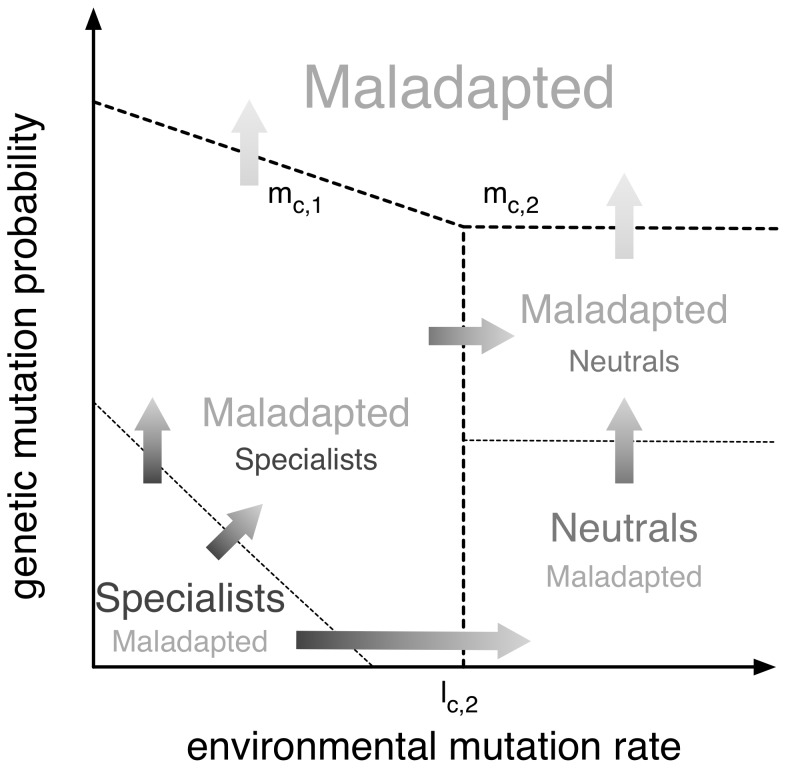
Phase diagram for the case 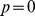
 **.** The most abundant genome is indicated by a larger name in each region. The average fitness of the population decreases along the arrows.

Fig. 4 sketches the phase diagram, as a function of 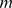 and 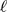, resulting from the previous equations. The different scenarios for small and large values of 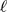 are now explicit. For small 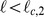, specialist individuals (in the 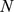 class) are able to survive, and even dominate the population, as long as the mutation rate is small. In fact, for 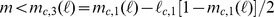, the density of specialists is larger than the density of maladapted individuals. For larger 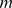, the density of specialized genomes decreases, until it reaches the 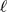-dependent threshold 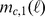, leading to a continuous, second order, phase transition (akin to the error catastrophe in quasispecies models [28]) beyond which the whole population becomes maladapted and thus prone to eventual extinction. In all of this region of small 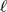, neutral individuals are irrelevant. For small 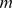, specialists perform much better, while for large 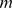 only maladapted individuals survive.

When 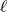 increases, a different picture emerges. For fixed, small 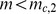, the explicit behavior of 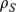 as a function of 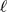, reads we can obtain from Eq. (6)

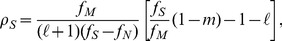
(10)for 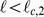, and zero otherwise. Thus, when crossing 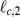, the density of specialized individuals experiences a first order transition to extinction, with a jump of magnitude
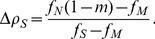
(11)The sudden extinction of specialized individuals coincides with the abrupt emergence of neutrals, in a related first order transition for 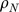 with an associated jump
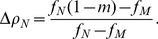
(12)In this large 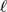 region, neutrals are able to cope with environmental change if the mutation rate is sufficiently small, again up to a maximum mutation rate 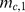, after which 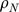 continuously becomes zero and only maladapted individuals can survive. These discontinuous transitions as a function of 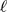 at fixed 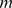 are reminiscent of the phenomenology observed in quasispecies models with higher order replication mechanisms [35]. We note, however, that transition as a function of 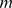 at fixed 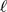 are all continuous.

**Figure 5:**
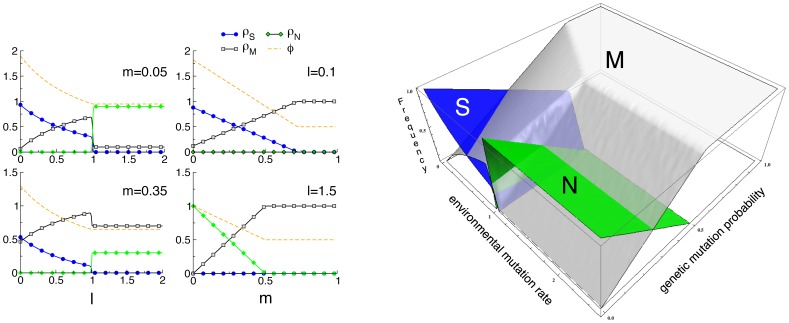
Species densities at the steady state for small 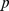
 **.** Left panel: Genome densities as a function of the environmental mutation rate 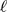 for fixed 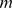, and as a function of 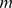 for fixed 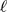. Fitness values are chosen as 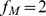, 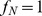 and 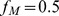. Hence, 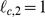, 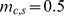 and 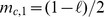, implying 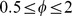 (see main text). Right panel: Densities of 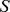 (blue), 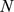 (green) and 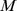 (gray) genomes as a function of the environmental mutation rate 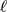 and the genetic mutation rate 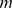.

Fig. 5 shows the proportions of the three genomes along with the average fitness as function of 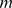 for fixed 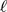, and as a function of 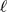 at fixed 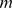 (left panel), and the general scenario as a function of both 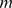 and 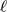 (right panel). As it is clear, while an abrupt transition occurs at the level of the genome frequencies at 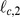 (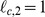 in Fig. 5), the average fitness 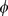 exhibits a continuous behavior, decreasing monotonously from the maximum 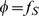 for 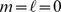 to 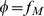 for large values of 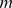 (when all the other genomes simply mutate into 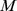). When 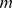 is fixed (left column, left panel), increasing 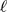 causes an increase of 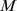 genomes and a simultaneous decrease in 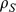 and 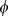. As 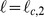, however 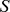 genomes disappear as the neutral genomes abruptly appear. The latter guarantees a constant value of 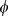. 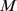 genomes are constantly created due to the genetic mutation rate, but their fitness is lower so they do not reproduce frequently. This scenario is stable, and any further increase in 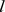 does not produce any effect. The role of 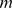 is better understood at fixed values of 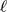. When 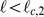 (top panel) increasing 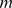 deteriorates the fitness of the population since 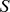 genomes are substituted by 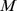 ones, which eventually become fixed (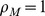 as 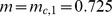 in figure). A similar behavior is observed for 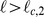 (bottom panel), but here the 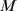 genomes take the place of the 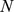 genomes, till the latter disappear at 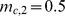 for the values of the simulations.

The crucial difference between the cases 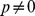 and 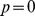, as can be observed from the comparison of Figs. 2 and 5 is the effect of a positive density of specialists for large 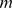 and 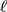. In the case 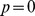, the density 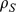 goes to zero after the corresponding transition, specialist being unable to cope with extreme genetic and/or environmental rates of change. In the presence of a non-zero 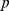, signaling the possibility of collateral beneficial effects of an environmental change to previously maladapted individuals, susceptible individuals are still able to thrive in an situation combining both fast genetic and environmental change (see lower right plots in Fig. 2). This effect is due to the feedback mechanism induced by the parameter 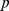, that allows the replenishment of the 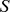 individuals from previously maladapted individuals. Their prevalence is however relatively small, and comparatively negligible with respect to the predominant species, either 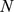 or 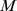, especially in the case of small populations. Finally, it is worth noting that, while the prevalence of 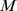 genomes is stable in our model, it can be interpreted as a metastable state leading to extinction in a multi-species scenario.

## Discussion

The model presented in this paper shows that a genetic adaptation to a specific form of an environmental feature is profitable only as long as the rate of change in the environment is not too fast. Indeed, a phase transition determines the onset of a different regime in which a neutral strategy is advantageous. The critical value of the environmental rate separating the two phases is proportional to the difference between the fitness of the neutral and specialist individuals. This analysis is the consequence of the simplicity of the model that, in contrast to previous modeling attempts, allows us not only to outline a qualitative scenario, but also to characterize quantitatively and in a transparent way the role of the different parameters, in the hope that future experimental work will be able to test these findings.

The analysis proposed here provides a framework for understanding a range of empirical finding. As mentioned above, for example, lactose tolerance did become genetically encoded [5] while language is a moving target for the genes, that appears to change too fast to allow genetic adaptation [8]. In the same way, agricultural practices that determine an increased presence of malaria are linked to genetic mutations that cause malaria reduction [36]–[38], and bioinformatic methods have recently shown that climate has been an important selective pressure acting on candidate genes for common metabolic disorders [39]. Another particularly significant example comes from biology, where the diversity and temporal variability of a population of hosts determines the pressure for parasites to specialize on one host or to become generalists on a wide range of hosts [40], as it has been experimentally shown for example in parasites *Brachiola algerae* infecting *Aedes aegypti* mosquitoes [26]. Our model coherently predicts also that specialized genomes would decrease their fitness if the mutation rate of the corresponding environmental feature increases (Fig. 5). Interestingly, this is what has been observed in relation to climate change, the consequence being a diminished robustness against competitors and natural enemies, which, in a multi-species scenario, could eventually lead to extinction [3].

In summary, we have introduced an evolutionary model that captures a wide array of natural scenarios in which genes evolve against a potentially changing environment. These results have been obtained using strong simplifying assumptions that can be relaxed in future work. For example, a natural extension of the model could consider a more complex network of environment-gene interactions, including the possibility of feedback between genes and the rate of change in the environment. Such a generalization could lead to important results and a richer phenomenology [41], [42], as well as enlarge the range of applicability of the model [43], even though it may reduce the mathematical tractability of the resulting equations. Likewise, the fitness of each genome could depend, for example, on its relative abundance in the population, instead of being a constant parameter. Finally, the equations we have derived apply in the case of very large populations; a possible extension could consider the effects of fluctuations in small groups. The framework we have put forth is general and allows these and other aspects (such as the effects of spatial fluctuations in finite dimensions) to be addressed in a principled way.
